# A novel lotus seed cross-linked resistant starch: Structural, physicochemical and digestive properties

**DOI:** 10.3389/fnut.2022.989042

**Published:** 2022-08-09

**Authors:** Lanxin Li, Shuqi He, Yongjie Lin, Baodong Zheng, Yi Zhang, Hongliang Zeng

**Affiliations:** ^1^College of Food Science, Fujian Agriculture and Forestry University, Fuzhou, China; ^2^Fujian Provincial Key Laboratory of Quality Science and Processing Technology in Special Starch, Fujian Agriculture and Forestry University, Fuzhou, China; ^3^China-Ireland International Cooperation Center for Food Material Science and Structure Design, Fujian Agriculture and Forestry University, Fuzhou, China

**Keywords:** lotus seed cross-linked resistant starch, cross-linking, structural properties, physicochemical properties, *in vitro* digestion

## Abstract

The structural properties and physicochemical characteristics of lotus seed cross-linked resistant starches (LSCSs; LS-0CS, LS-1CS, LS-2CS, LS-4CS, LS-6CS, LS-8CS, LS-10CS, and LS-12CS) with different concentrations of cross-linking agents were investigated. The degrees of cross-linking of LSCSs increased along with the amount of cross-linking agent. The higher the degree of cross-linking, the greater the degree of LSCSs granule agglomeration. The occurrence of the cross-linking reaction was confirmed by the appearance of P = O at 1,250 cm^–1^ as assessed by FT-IR, and the covalent bonds formed by the phosphate group in LSCSs were mainly composed of distarch monophosphate (DMSP) as determined by ^31^P NMR. As the crosslinking degree increased, the peak strength of DMSP in starch was stronger and the specific gravity of DMSP was larger. Among the samples, LS-12CS had the highest cross-linking degree, with a greater specific gravity of DMSP. Moreover, the solubility levels of LSCSs decreased and the thermal stability and anti-digestive properties improved as the cross-linking degree increased, which was correlated with the degree of agglomeration and DMSP in LSCSs. The RS content of LS-12CS was 48.95 ± 0.86%.

## Introduction

Lotus seeds are an economically important specialty product in China, with a greater than 2,000-year history of cultivation. Starch, the main constituent of lotus seeds, represents approximately 50% of the dry basis and is abundant in raw material resources, especially during the processing of lotus seed milk drinks that produce numerous lotus seed starch by-products (1). Natural starches are susceptible to reconstitution and dehydration during storage, low resistant to pH, temperature changes and mechanical treatment and have unstable functional properties. However, these inherent functional limitations can be overcome by starch modification. Resistant starch (RS) is divided into five types: physically trapped starch (RS1), natural resistant starch granules (RS2), retrograded starch (RS3), chemically modified starch (RS4) and a complex of amylose and lipids (RS5). They all require modifications to form, except for RS1 and RS2 (2). These starches are prepared using three main methods: physical, chemical and enzymatic modifications.

RS4 is currently being widely studied and applied in routine food products. It is formed using chemical modification methods, such as etherification, esterification and cross-linking reactions. Cross-linking treatments are usually used in the chemical modification of starch. During the cross-linking reaction, cross-linking agents are introduced into the intermolecular bridges between biopolymer layers to undergo chemical reactions, such as esterification reaction and etherification, which may change the overall structural properties and surface chemistry of the polymer backbone (3). The cross-linking agents commonly used in this reaction are sodium trimetaphosphate (STMP), sodium tripolyphosphate (STPP), epichlorohydrin and phosphoryl chloride (4). Phosphoryl chloride reacts violently with water and poses certain safety hazards. Although epichlorohydrin has the advantages of mild reaction, easy control and excellent cross-linking effect, it is toxic by oral and nasal inhalation and skin absorption. STMP and STPP are nontoxic and safe with low costs, controllable degrees of substitution, ability to change physical and chemical properties even at low substitution, and small structural changes of starch chain bases. The cross-linking reaction results in the starch structure being strong and dense, reduces the transparency of the paste, increases the resistance of the starch granules to acid, heat and shear, and reduces their tendency to dissolve and swell (5). Sandhu et al. (6) prepared cross-linked sorghum starch with different doses (0.1–1.0%) of epichlorohydrin as cross-linker and showed that its amylose content, swelling power and solubility decreased as the amount of cross-linker increased compared with raw starch. The surface of cross-linked sorghum starch is rough, with cavities and cracks. Broad bean starch with different concentrations (1, 3, and 5%) STMP as a cross-linking agent was formed by Sharma et al. (7). The cross-linked starch has a higher phase transition temperature and enthalpy of pasting (ΔH) compared with the raw starch, indicating a denser internal structure of the starch granules. The content of RS increases along with the concentration of cross-linking agent, indicating limitations in the actions of digestive enzymes.

Although much research has been conducted on the preparation and characterization of cross-linked starches, it is meaningful to study their structure-phosphorylation-function relationships in depth. No studies on the modification of lotus seed starch by cross-linking have been performed. Thus, here, lotus seed starch was reacted with different doses of cross-linking agents (STMP/STPP) in an alkaline environment to form lotus seed cross-linked resistant starches (LSCSs) with different degrees of cross-linking. The aim was to characterize their structural properties and to investigate the physicochemical properties as well as *in vitro* digestibility of LSCSs prepared by the addition of different cross-linking agents.

## Materials and methods

### Materials

Lotus seed starch (Green Field Food Co., Ltd., Fujian, China) was isolated as described previously (8). Sodium trimetaphosphate (STMP) and sodium tripolyphosphate (STPP) were obtained from China National Pharmaceutical Group Chemical Reagent Co. The α-amylase and glucose amylase for *in vitro* digestibility studies were provided by Shanghai Yuanye Biotechnology Co.

### Preparation of lotus seed cross-linked resistant starch

LSCSs was prepared using the methodology of Carmona-Garcia et al. (9) with slight modifications. Lotus seed starch (20 g) was dispersed in water (30 mL), mixed with Na_2_SO_4_ (2 g), and the pH adjusted to 11.5 using NaOH (1 mol/L). Then, 0%, 1%, 2%, 4%, 6%, 8%, 10%, and 12% (w/w) of mixed cross-linking reagents (STMP/STPP, 99:1) were added independently, and stirred at 50°C for 4 h in a water bath. Then, HCl (1 mol/L) was used to adjust the pH to 6.5, and the starch slurry was washed with distilled water until neutralization. Samples were centrifuged two to three times at 3,000 × *g*, then hot air-dried at 45°C for 24 h. These samples were designated as LS-(0, 1, 2, 4, 6, 8, 10, and 12) CS, respectively.

### Degree of cross-linking

**FIGURE 1:**
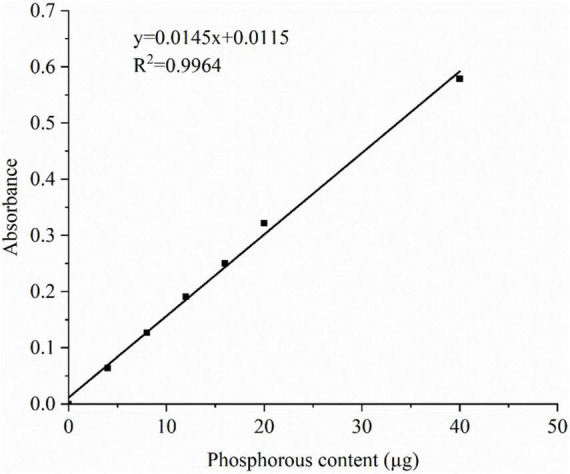
Standard curve of the phosphorus content.


The total phosphorous content of each cross-linked starch was determined as previously described (10). The samples (0.5 mg) were digested with a mixture of concentrated nitric acid and concentrated sulfuric acid. The absorbance of the solution at 825 nm was determined spectrophotometrically. The absorbance of the measured solution was compared with the calibration curve to determine the phosphorous content, and the phosphorous content was calculated using Eq. (1). The phosphorous content was used to express the degree of cross-linking. Standard curve of the phosphorus content is shown in Figure 1

(1) $$Phosphorouscontent\left(\%\right)=\frac{M_{1}\times V_{0}\times 100}{M_{0}\times V_{1}\times 10^{6}}$$

where *M*_1_ (μg) represents the phosphorus content of the sample solution determined from the standard curve, *V*_0_ (mL) represents the volume of the sample solution, *M*_0_ (g) represents the mass of the test sample and *V*_1_ (mL) represents an equal volume of sample solution for determination.

### Scanning electron microscopy (SEM)

Scanning electron micrographs of starch samples were obtained by SEM (PHILIPS = XL30 ESEM, Philips-FEI, Netherlands) with a magnification of 5,000× operating at an accelerating voltage of 6.0 kV, as described by Lin et al. (11) with some modifications. A small amount of sample was uniformly distributed on the conductive adhesive metal platform and gold sprayed.

### X-ray diffraction

X-ray diffraction patterns and relative crystallinities of the samples were determined using an X-ray diffractometer (Xpert 3; Analyx Corp. Ltd., Boston, MA, United States), as described by Zhang et al. (1), with some modifications. The diffraction angle was scanned from 5°to 50°at a scanning rate of 5°/min, with a target voltage of 40 kV and a current of 200 mA. The degrees of crystallinity were calculated by Eq. (2).


(2) $$Crystallinity\left(\%\right)\frac{\text{Ac}}{\text{Aa}+\text{Ac}}\times 100\%$$

where *Ac* represents the crystalline area on the X-ray diffractogram, and *Aa* represents the amorphous area on the X-ray diffractogram.

### Fourier transform infrared (FT-IR) spectroscopy

The FT-IR spectra of the samples were measured using an FT-IR spectrometer (Avatar 360, Thermo Nicolet Corporation Ltd., Madison, WI, United States). The starch samples were mixed well with dried KBr at a ratio of 1:100, ground in an agate mortar under an IR lamp and pressed into round thin slices. The scanning wave number range was 400–4,000 cm^–1^ with a resolution of 4 cm^–1^ and 32 scans.

### ^31^P NMR spectroscopy

^31^P NMR spectra were obtained using an NMR spectrometer (Avance III 500, Bruker Ltd., Karlsruhe, Germany) by solid state resonance, as described by Dong & Vasanthan (2), with some modifications. Pure solid samples were analyzed without any treatment. The spectrometer was configured for a frequency analysis of 161.67 MHz at room temperature, using a double-resonance probe with a CP/MAS detection system.

### Swelling power and solubility

Swelling power and solubility were measured in accordance with a modified method of Miaomiao (12). The samples (0.4 g) were mixed with water (40 mL), heated, and stirred in a 95°C water bath for 30 min. They were then cooled to room temperature, centrifuged at 3,000 × *g* for 30 min, and the weights of the dissolved pellet and the supernatant after complete evaporation were directly measured. Swelling power and solubility were calculated using Eqs. (3) and (4), respectively.


(3) $$\text{Solubility}\left(\%\right)=\frac{\text{A}}{\text{W}}\times 100\%$$

(4) $$\mathrm{Swelling}\,\mathrm{power}\,\left(g/g\right)=\frac{\text{P}}{\text{W}}\,\left(1-\text{Solubility}\right)$$

where *W* (g) represents the weight of a starch sample; and *A* and *P*(g) represent the weights of the dried supernatants and swollen granules respectively

### Differential scanning calorimetry (DSC)

The thermal properties of the samples were measured using a differential scanning calorimeter (DSC-Q2000 TA Instruments, New Castle, DE, United States), as described previously (13). Thermal parameters, including melting enthalpy (*ΔH*), onset temperature (*T*_*o*_), peak temperature (*T*_*p*_) and conclusion temperature (*T*_*c*_), were recorded.

### *In vitro* starch digestibility

The *in vitro* digestibility of starch was analyzed in accordance with the method of Zheng et al. (14) with some modifications. The samples (0.2 g) were dispersed in sodium acetate buffer (10 mL, pH = 5.2) and incubated at 37°C for 20 min. Then, a mixture (10 mL) of α-amylase (200 U/mL) and amyloglucosidase (160 U/mL) was added and incubation continued in a 37°C water bath with shaking (100 rpm/min). At different time points (0, 20, 40, 60, 90, 120, 150, and 180 min) starch digest (0.5 mL) was aspirated and mixed with 2 mL of anhydrous ethanol to inactivate the enzymes, followed by the addition of distilled water (4 mL) and dilution to 5 mL for the determination of sugar content using the glucose oxidase method. The hydrolysis rate, rapidly digestible starch (RDS), slowly digestible starch (SDS) and resistant starch (RS) contents were calculated using Eqs. (5), (6), (7), and (8), respectively.


(5) $$Hydrolysisrate\left(\%\right)=\frac{\text{Gt}}{\text{TS}}\times 0.9\times 100\%$$

(6) $$RSD\left(\%\right)=\frac{G_{20\,m\,i\,n}-G_{0\,m\,i\,n}}{\text{TS}}\times 0.9\times 100\%$$

(7) $$SDS\left(\%\right)=\frac{G_{120\,m\,i\,n}-G_{20\,m\,i\,n}}{\text{TS}}\times 0.9\times 100\%$$

(8) RS(%)=(1-RDS-SDS)×100%

where *Gt* represents the glucose release from hydrolysis at time *t*, *G*_0min_ represents the free glucose content at 0 min of digestion, *G*_20min_ represents the glucose release at 20 min of digestion, *G*_120min_ represents the glucose release at 120 min of digestion, and *TS* represents the weight of the starch sample.

### Statistical analysis

All the experiments were repeated at least three times. Experimental graphics were processed using Origin 9.0 (OriginLab Corporation, Northampton, MA, United States). Data were analyzed and statistical significances were determined using DPS 9.5 (Science Press, Beijing, China) (*p* < 0.05).

## Results and discussion

### Crosslinking degree of lotus seed cross-linked resistant starch

**FIGURE 2:**
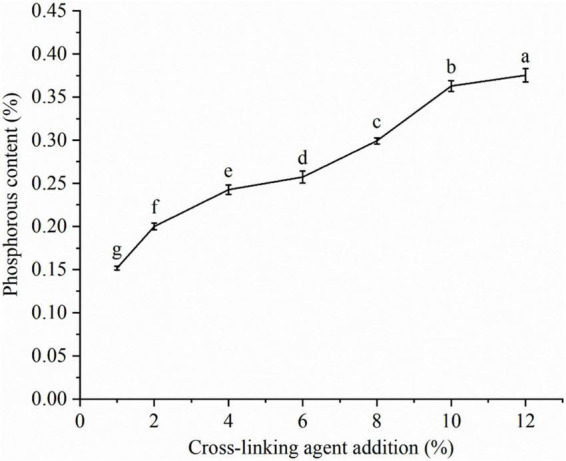
Phosphorous content of lotus seed cross-linked resistant starch. Different lowercase letters represent significant differences (*p* < 0.05).

The effect of cross-linking agent (STMP/STPP) addition on the degree of cross-linking of LSCSs is shown in Figure 2. The combined phosphorous content showed an obvious increasing trend along with the increased addition of cross-linking agent. When the cross-linking agent content was 12%, the amount of combined phosphorus in the starch was 0.38% ± 0.008%, which did not exceed the upper limit for food safety (0.4%) (15). The effects of different cross-linking agent additions on the crosslinking degree in three crystal forms (A, B and C) of starch have been investigated by Kou and Gao (16). They demonstrated that the phosphate group of STMP/STPP molecules covalently bound with the ionized hydroxyl group of the anhydroglucose unit of starch. The phosphorus content is a value that indirectly indicates the degree of cross-linking. This is because as the amount of cross-linking agent added increases, the more phosphate groups of STMP/STPP molecules involved in the cross-linking reaction, the more phosphorus content in the generated products (17). LSCSs had the highest degree of cross-linking when the cross-linking agent content was 12%. This was in agreement with the results of Sharma et al. (7). They cross-linked broad bean starch at different levels (1, 3, and 5%) using STMP as a cross-linking agent and found that the degree of cross-linking increases along with the cross-linking agent’s level.

### Scanning electron microscope of lotus seed cross-linked resistant starch

**FIGURE 3:**
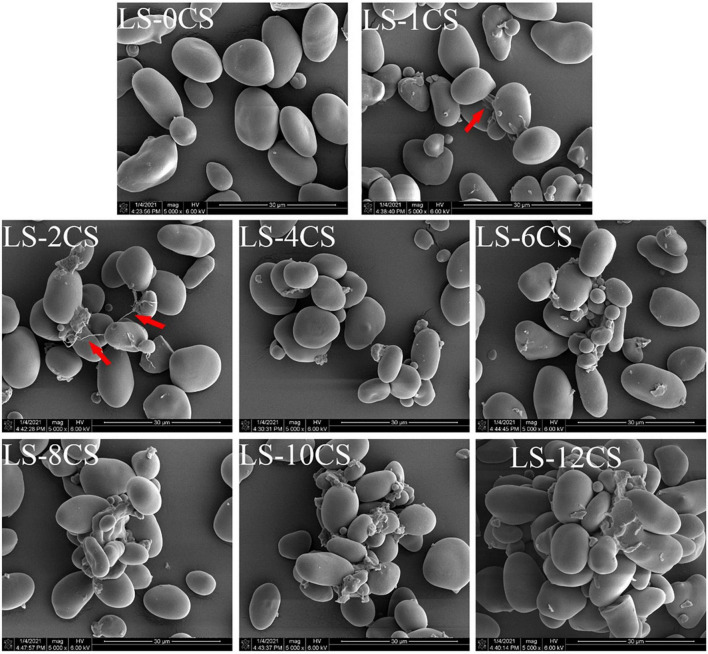
Scanning electron microscope images of lotus seed cross-linked resistant starch samples.

Scanning electron micrographs of LSCSs are shown in Figure 3. The starch granule structure of LS-0CS was complete, circular or oval in shape, with a smooth surface, whereas those of other LSCSs did not change, which indicated that the cross-linking reaction did not change their granular morphology. The granular morphology of LS-0CS was consistent with that of natural lotus seed starch (1). However, adhesion between the starch granules of LS-1CS and LS-2CS was clearly observed owing to the crosslinking reaction between the starch and the cross-linking agent (See the arrow), which resulted in the formation of a “bridge”, and promoted adhesion, between the starch molecules. The phenomenon in the present study was consistent with the results reported by Chen et al. (18), in which cross-linked starches were prepared by cross-linking kudzu starch with STMP. They found that starches with different degrees of cross-linking were all similar to the raw starch in terms of granular morphology, and granular adhesion was also observed. Here, the starch granules of LS-4CS started to agglomerate owing to the adhesion phenomenon, and the degree of agglomeration between LSCSs starch granules became stronger as the degree of cross-linking increased, which may result from the introduction of cross-linking agent. Functional groups change the intermolecular interactions between starch chains and connect starch molecules to form spatial network structures. More functional groups were introduced as the cross-linking degree increased, which resulted in more starch molecules being connected and a tighter spatial network structure. Consequently, the degree of agglomeration was greater.

### Crystal structure analysis of lotus seed cross-linked resistant starch

On the basis of the source, a natural starch crystal structure is categorized as A, B, C or V type. The characteristic diffraction peaks of the crystalline structure of A-type are at 15°, 17°, 17.8°, and 23°(2θ), and the characteristic diffraction peaks of the crystalline structure of B-type are at 5.6°, 17°, 19.5°, 22°, and 24° (2θ) (19). The crystalline structure of C-type is a mixture of A-and B-crystalline structures, containing characteristic peaks of both crystalline structures (20). Previous studies have shown that natural lotus seed starch is a C-type crystal structure with main diffraction peaks at 14.93°, 17.03°, and 23.02° (2θ) (1).

**FIGURE 4:**
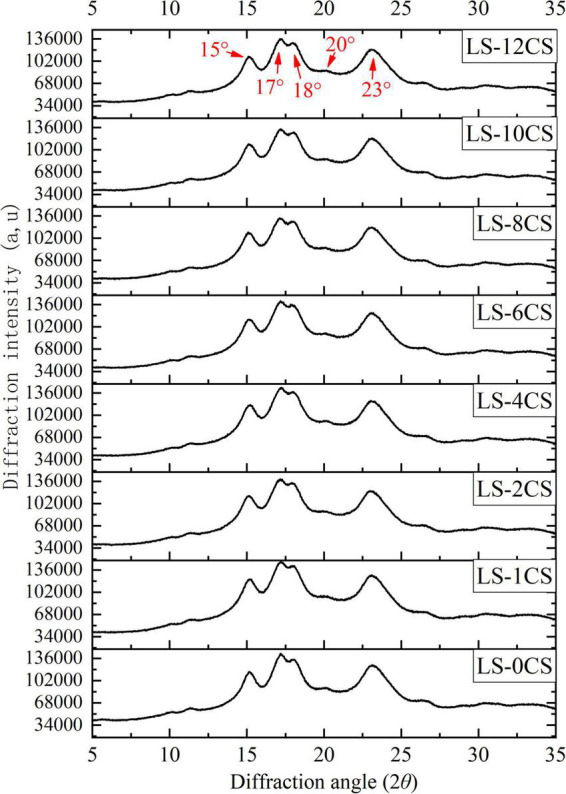
X-ray diffraction patterns of lotus seed cross-linked resistant starch samples.

The X-ray diffraction pattern of LSCSs is shown in Figure 4. All the starch samples had obvious diffraction peaks near 15°, 17°, 18°, and 23° (2θ), and a weak diffraction peak at 20° (2θ), which indicated that all the starch samples contained characteristic peaks of the crystalline structures of A- and B-types, making them C-types. This was in accordance with the results of a previous study (1). The positions of the main X-ray diffraction peaks of LSCSs were not affected by the cross-linking reaction compared with LS-0CS, which did not change with increasing degree of cross-linking, because the cross-linking agent acted as a bridge to shorten the distance between starch molecules without changing the crystallization structure within the starch granules. Additionally, the cross-linking reaction mainly occurred in the amorphous regions on the starch granules that were easily exposed to the cross-linking agent. Thus, the cross-linking reaction did not change the crystal type of the starch granule. This conclusion is consistent with a previous report by Yang et al. (21). Moreover, Falsafi et al. (22) prepared cross-linked starches at different pH values (9–12), different cross-linker concentrations (STMP/STPP 99:1, 5–15%) and under sonication and conventional treatment conditions. They found that the cross-linking reaction did not affect the crystal type of the corn starch compared with the raw starch, which was further evidence that the cross-linking reaction occurred mainly in the amorphous region of the granule structure.

The relative crystallinity of LSCSs samples are shown in Table 1. Compared with LS-0CS, the crystallinity of the rest of LSCSs was significantly reduced. The crystallinity levels of LS-10CS and LS-12CS were the lowest, which indicated that the crystallinity decreased as the cross-linking increased, presumably due to chemical modifications, and that crystal structure changes occurred during the cross-linking process. The disorder of chain arrangements was caused by substituting the hydroxyl group of starch with phosphate, but the disruption of the crystalline region was not great enough to change the crystalline shape of starch because of the low cross-linking degree. These results were consistent with the previous results reported by Chen et al. (18), which showed that compared with the native starch, the crystallinity of cross-linked starch decreased.

**TABLE 1:** Relative crystallinity levels of lotus seed cross-linked resistant starch samples.

Sample	LS-0CS	LS-1CS	LS-2CS	LS-4CS	LS-6CS	LS-8CS	LS-10CS	LS-12CS
Relative crystallinity (%)	32.80 ± 1.13^a^	30.03 ± 0.95^b^	29.73 ± 1.12^bc^	29.63 ± 0.64^bc^	29.47 ± 0.55^bc^	29.10 ± 0.42^bc^	28.20 ± 0.57^c^	28.15 ± 0.21^c^

Different superscript letters indicate significant differences (*p* < 0.05).

### Fourier transform infrared (FT-IR) spectroscopic analysis of lotus seed cross-linked resistant starch

**FIGURE 5:**
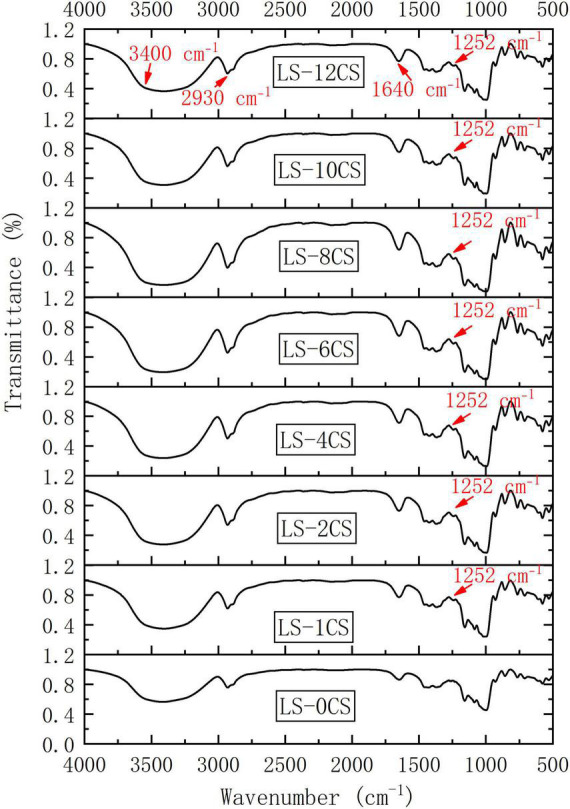
FT-IR spectroscopy of lotus seed cross-linked resistant starch samples.

The variations in helical structures, chain conformations and crystal forms of starches alter their absorption of infrared energy. Therefore, the changes in the molecular structure of different LSCSs were analyzed using FT-IR spectroscopy, as shown in Figure 5. The extreme broad band at approximately 3,300–3,500 cm^–1^, which was attributed to inner- and intro-hydroxyl group stretching vibration, was observed in all the starches. The band at 2,930 cm^–1^, which was strong and sharp, represents the anti-symmetry stretching vibration of the carbon-hydrogen bond (23). Because of the H_2_O bending vibration, and given that the hydroxyl groups in water molecules were absorbed by starch, the absorption at 1,640 cm^–1^ is a characteristic absorption band of starch. The shapes and positions of the spectral peaks of all the LSCSs were almost the same. The molecular structures of the samples remained unchanged after the cross-linking treatment, but further observations revealed that the degree of O-H stretching vibration near 3,400 cm^–1^ of the LSCSs changed and the intensity of the peak weakened compared with LS-0CS. This indicated that the alcoholic hydroxyl group of the starch was covalently bound to the phosphate group of the crosslinking agent. This result was in accordance with the reports of Xie et al. (4). A new peak at 1,250 cm^–1^ appeared in the LSCSs, and it was characteristic of P = O bonds in crosslinked starch, which confirmed that the starch samples had reacted with STMP/STPP. This was consistent with the results reported by Shalviri et al. (24). Moreover, when Ashwar et al. (23) prepared rice crosslinked starch, the appearance of P = O at 1,244 cm^–1^ was also observed by FT-IR spectroscopy.

### ^31^P NMR of lotus seed cross-linked resistant starch

**FIGURE 6:**
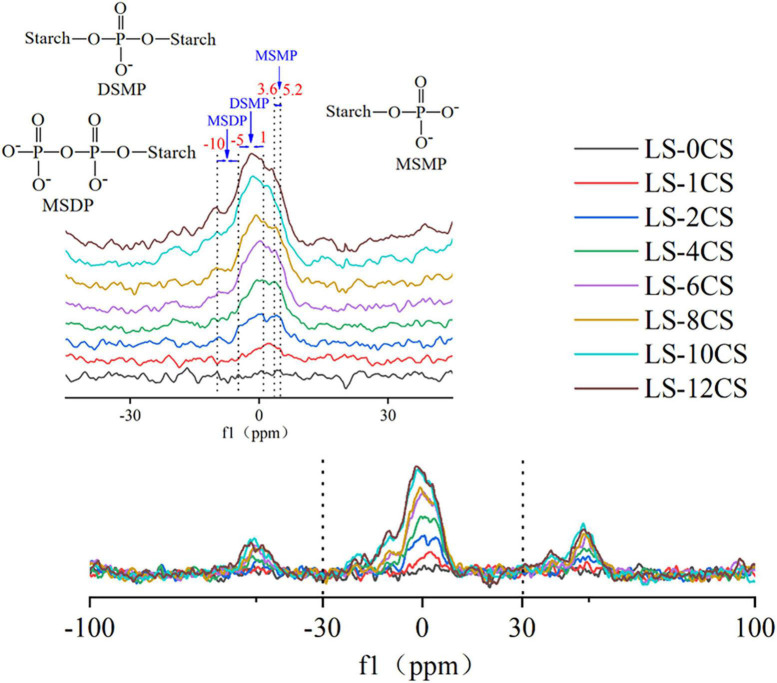
^31^P NMR of lotus seed cross-linked resistant starch samples.

During the reaction of starch with cross-linking agents, the starch phosphate is formed (25). The starch phosphate content was adjusted by controlling the degrees of starch esterification and crosslinking, which in turn improved the functional properties of the starch. The ^31^P NMR spectra of lotus seed cross-linked resistant starches are shown in Figure 6. The signal peaks at -10 ppm to -5 ppm, -5 ppm to 1 ppm, and 3.6 ppm to 5.2 ppm were attributed to the structures of monostarch diphosphate (MSDP), distarch monophosphate (DSMP) and monostarch monophosphate (MSMP), respectively, as studied previously (5).

The LSCSs had different signal peaks compared with LS-0CS, indicating phosphate starch ester generation, which further confirmed the occurrence of the cross-linking reaction. This was consistent with the results of the FT-IR spectroscopy. LS-1CS and LS-2CS had weak signals at 4.7 ppm, and LS-4CS, LS-6CS and LS-8CS had weak signals at 3.6 ppm. The signal peak of MSMP was hardly observed in LS-10CS and LS-12CS. In the other LSCSs, a small amount of MSMP production occurred. Moreover, the MSMP signals at 4.7 ppm, 4.4 ppm, and 3.6 ppm correspond to the phosphorylation of hydroxyl groups at C-2, C-3, and C-6, respectively (26). Therefore, the MSMP produced in LSCSs was mainly located at C-6 and C-3 of the glucose unit. This indicated that most of the cross-linkages formed in MSMP were between two glucose residues located on different starch chains, and a small portion of cross-linkages may have formed between two glucose residues in the same starch chain. Presumably, because MSMP was unstable, LS-10CS and LS-12CS did not produce signal peaks in the range of 3.6 ppm to 5.2 ppm, which was consistent with the previous results reported by Sang et al. (27), who found that after stirring wheat 10.5 in a slurry at pH 11.5 for 3 h at 45°C, MSMP signal peaks of wheat starch with a high degree of cross-linking disappeared completely.

The LSCSs, except LS-1CS and LS-2CS, generated weak signals in the range of -10 ppm to -5 ppm, indicating that a small amount of MSDP was generated. During the reaction of starch with cross-linker (STMP/STTP) under alkaline conditions, the ring-opening attack of starch alcoholate ions on the cross-linker first formed mono-amyl triphosphate, which was unstable during the synthesis reaction. It had four negatively charged ionized hydrogens, and all three phosphoryl groups on its triphosphoryl chain had a strong ionizable acidic OH. When hydroxide or starch alcoholate ions react with monostarch triphosphate, pyrophosphate is more likely to detach from the group than orthophosphate, which leads to the reaction of an RO- or OH- ion with the α-phosphorous of the triphosphonyl group to form DSMP or MSMP, respectively. With a further reaction, the γ-phosphate group exfoliates, which in turn generates MSDP. Thus, when a small amount the cross-linking agent was added, the cross-linking reaction was not strong enough to generate MSDP.

The LSCSs had strong signals in the range of -1 to 0 ppm and a weak signal in the range of 0 to 1 ppm, which indicated that the cross-linking reaction mainly produced DSMP. A stronger signal in the -1 to 0 ppm range occurred as the degree of cross-linking increased, leading to a greater content of DSMP. Because the hydroxyl groups at C-6 and C-3 were more reactive to the cross-linking reagent than those at C-2, DMSP may only occur within the hydroxyl groups at C-6 and C-3. Lack et al. (28) found that the DSMP signal in the range of -1 to 0 ppm indicated a cross-linking reaction between two different starch molecules, and the DSMP signal in the range of 0 to 1 ppm was a cross-linking reaction within the starch molecule, meaning between the hydroxyl groups of the glucose part of the same chain. This indicated that the generation of DMSP in this study was mainly a cross-linking reaction between the lotus starch molecules. Thus, the cross-linking reaction under alkaline conditions could induce the existing MSDP and MSMP to form new cross-linked ester bonds with hydroxyl groups, increasing the DSMP level. Moreover, as shown in Figure 5, as the crosslinking degree increased, the peak strength of DMSP in starch was stronger and the specific gravity of DMSP was larger. The covalent bonds formed by phosphate groups are mainly composed of DMSP (29), and combined with the phosphorous spectral analysis in this study, DMSP was presumably closely related to the physicochemical and digestive properties of the LSCSs.

### Swelling power and solubility of lotus seed cross-linked resistant starch

**FIGURE 7:**
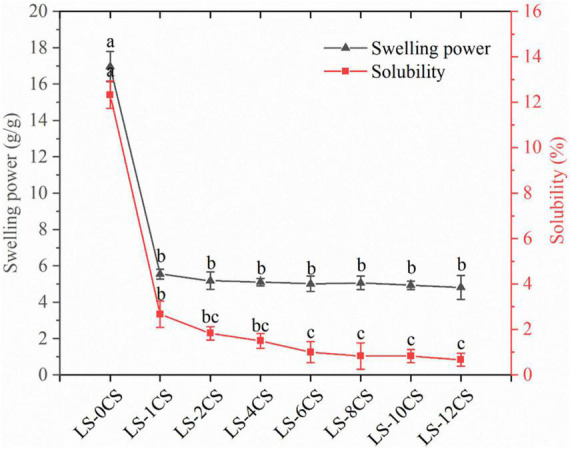
Swelling powers and solubility levels of lotus seed cross-linked resistant starch samples. Different lowercase letters represent significant differences (*p* < 0.05).

The swelling power and solubility indicate the magnitude of starch chain interactions within the amorphous and crystalline regions, respectively (30). The swelling power of starch granules is the characteristic of a disordered crystalline region and consequent association between hydroxyl groups and water molecules via hydrogen bonding. The solubility levels and swelling powers of the LCSCs under heating treatment are shown in Figure 7. Compared with LS-0CS, the solubility of the LSCSs were all significantly lower, with LS-6CS, LS-8CS, LS-10CS, and LS-12CS having significantly lower solubility levels than LS-1CS. Compared with LS-0CS, the crystallinity of the LSCSs were also all significantly lower. This indicated that the decrease in solubility was related to crystallinity. These results were consistent with the previous results reported by Chen et al. (18) in which the decrease in crystallinity contributes to the decrease in starch granule solubility. According to the ^31^P NMR analysis, the higher cross-linked LSCSs contained more phosphate groups forming covalent bonds, and the specific gravity of DMSP was greater. Additionally, the starch molecules were strengthened by covalent bonding, which caused a significant decrease in solubility. Shi et al. (12) prepared pea cross-linked starch using different concentrations of cross-linking agents (STMP/STPP, 99:1, w/w). They found that the solubility of pea cross-linked starch was lower than that of natural starch and that it decreased as the amount of cross-linking agent added increased.

The swelling power of LSCS was also significantly lower than that of LS-0CS because the cross-linking reaction enhanced the intra- and intermolecular bonds between amylose and amylopectin, increasing the density of starch by binding the starch chains together, decreasing the mobility of the macromolecular chains and the degree of decomposition of starch granules during pasting, which limited the expansion of starch granules. However, there were no significant differences among the swelling powers of the LSCSs, indicating that within a certain range, the leaching of starch molecules from the starch granules was not significantly affected owing to the increase in cross-linking. Because the hydrogen bond in starch was broken during the pasting process and replaced by hydrogen bond with water, the FT-IR spectroscopy showed that the peak of LS-0CS located at 3,400 cm^–1^ was related to the hydroxyl stretching vibration of free and hydrogen-bonded hydroxyl groups, and the cross-linking reaction weakened the hydrogen bond during the pasting process, which was not easily replaced by the hydrogen bond of water. Thus, the starch granules remained stable and did not easily swell. These results agreed with that of Chen et al. (18). They cross-linked kudzu starch with STMP at different temperatures to obtain cross-linked kudzu starches with different degrees of cross-linking, and the resulting starches’ swelling powers were significantly lower than that of the original starch. Additionally, the swelling power did not change significantly as the degree of cross-linking increased.

### Thermal stability of lotus seed cross-linked resistant starch

The thermodynamic parameters of the LCSCs are shown in Table 2. Compared with LS-0CS, the phase transition temperatures of the LSCSs increased, with the T_0_ of LS-12CS being significantly higher than those of LS-1CS, LS-2CS, LS-4CS, and LS-6CS, and the T_*c*_ and T_*p*_ values of LS-6CS, LS-8CS, LS-10CS, and LS-12CS being significantly higher than those of LS-1CS, LS-2CS, and LS-4CS. This indicated that higher temperatures were required for LSCSs pasting owing to the covalent bonds formed by phosphate groups in the LSCSs that strengthened the bonding between starch molecules to different degrees. Additionally, the cross-linking treatment inhibited the melting of crystals in starch granules and tightened their lattice structures, which increased the pasting temperature. As indicated by the ^31^P NMR analysis, the covalent bonds formed by phosphate groups were mainly composed of DMSP (29). The specific gravity of DMSP increased along with the degree of cross-linking; therefore, LS-12CS had the highest phase transition temperature and required the highest pasting temperature. The transition temperature range of starch (T_*c*_–T_0_) is a measure of the integrity of the microcrystalline structure of amylopectin. The higher the values of T_*c*_–T_0_, the wider the heat absorption peak (31). Compared with LS-0CS, the T_*c*_–T_0_ values of the LSCSs were significantly higher, with the T_*c*_–T_0_ values of LS-12CS being significantly higher than those of LS-1CS and LS-2CS, which implied that the amylopectin microcrystals were more uniform after cross-linking treatment. These results were consistent with the study of Dong and Vasanthan (32), in which broad bean cross-linked and pea cross-linked starches were prepared, and the DSC showed that the T_0_, T_*p*_, T_*c*_, and T_*c*_–T_0_ values of the natural bean starch were significantly lower than those of the cross-linked starch.

**TABLE 2:** Thermal stability levels of lotus seed cross-linked resistant starch samples.

Sample	T_*o*_ (°C)	T_*p*_ (°C)	T_*c*_ (°C)	T_*c*_-T_*o*_ (°C)	Δ H_1_ (J/g)
LS-0CS	67.71 ± 0.16^d^	74.58 ± 0.10^e^	79.40 ± 0.21^c^	11.70 ± 0.37^c^	16.41 ± 0.03^a^
LS-1CS	68.12 ± 0.18^cd^	76.29 ± 0.14^d^	81.70 ± 0.16^b^	13.59 ± 0.02^b^	16.28 ± 0.04^a^
LS-2CS	68.27 ± 0.11^bcd^	76.57 ± 0.11^c^	81.90 ± 0.16^b^	13.63 ± 0.04^b^	14.36 ± 0.07^b^
LS-4CS	68.42 ± 0.15^bc^	76.96 ± 0.07^b^	82.11 ± 0.10^b^	13.70 ± 0.05^ab^	13.71 ± 0.06^c^
LS-6CS	68.51 ± 0.21^bc^	77.38 ± 0.13^a^	83.04 ± 0.22^a^	14.23 ± 0.01^ab^	13.18 ± 0.05^d^
LS-8CS	68.72 ± 0.42^abc^	77.41 ± 0.08^a^	83.09 ± 0.23^a^	14.37 ± 0.65^ab^	10.65 ± 0.06^e^
LS-10CS	68.82 ± 0.40^ab^	77.51 ± 0.12^a^	83.20 ± 0.17^a^	14.38 ± 0.57^ab^	10.59 ± 0.04^e^
LS-12CS	69.19 ± 0.25^a^	77.52 ± 0.16^a^	83.42 ± 0.13^a^	14.53 ± 0.27^a^	10.31 ± 0.10^f^

Different superscript letters in the same column indicate significant differences (*p* < 0.05).

The energy required for the dissociation of the crystalline double helix is represented as ΔH. Compared with LS-0CS and LS-1CS, the ΔH values of the remaining LSCSs were significantly lower and the ΔH decreased significantly as the degree of cross-linking increased. This indicated that less thermal energy was required to melt the double helixes of the LSCSs, probably due to the more complex and amorphous internal structures of cross-linked starches. These starch samples were not only connected by hydrogen bonding but also molecularly connected by cross-linking reactions, which was consistent with the change in the relative crystallinity calculated by X-ray diffraction. Similar findings were determined in cross-linked starches from common maize, broad beans and field peas (32), in which the cross-linking reaction increased the thermal stability, broadened the value of T_*c*_–T_0_ and decreased the ΔH value to varying degrees compared with natural starches.

### *In vitro* digestion characteristics of lotus seed cross-linked resistant starch

**FIGURE 8:**
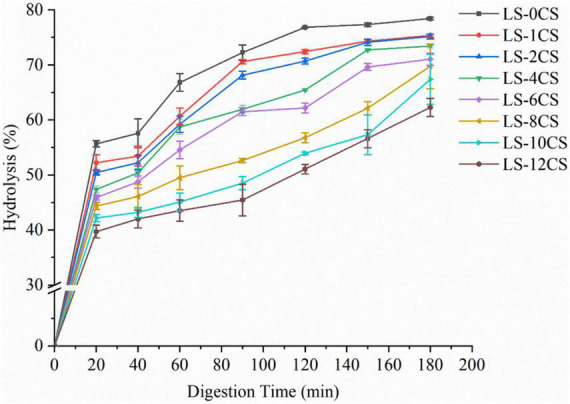
Hydrolysis rates of lotus seed cross-linked resistant starch samples.

The hydrolysis curves and digestion properties of the LSCSs are shown in Figure 8. Under the same digestion conditions, the hydrolysis rates of the LSCSs were lower than that of LS-0CS, with the most significant effect on LS-12CS after 120 min of digestion. This indicated that the hydrolysis effect of digestive enzymes on starch granules was significantly reduced by the cross-linking reaction, resulting in their lower digestibility levels. The hydrolysis patterns of the LSCSs per unit time showed that their digestibility decreased to different degrees with the length of the cross-linking reaction. This indicated that some of the hydroxyl groups of starch were replaced by phosphate ester bonds generated by the cross-linking reaction, and the presence of phosphate groups on the starch chains sterically hindered the formation of starch-amylase complexes. The abilities of digestive enzymes to enter the internal structures of starches were enhanced by the swelling of starch granules; consequently, the limited swelling of cross-linked starch inhibited its hydrolysis rate. Moreover, the SEM analysis revealed that the higher the degree of cross-linking, the stronger the agglomeration of starch granules, which to a certain extent inhibited the digestive enzymes. This was consistent with the results of Shi et al. (12), who cross-linked starch with different concentrations of STMP and STPP (99:1, w/w) and showed that the higher the degree of cross-linking, the more resistant the starch was to digestion by enzymes. This may also be related to various other factors, such as solubility and swelling power.

**FIGURE 9:**
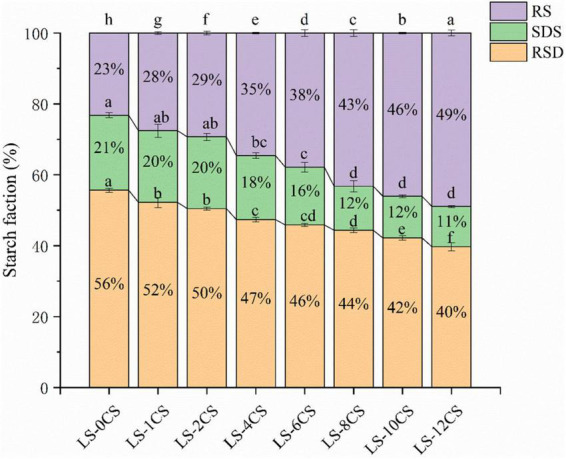
RDS, SDS, and RS contents of lotus seed cross-linked resistant starch samples. Different lowercase letters represent significant differences (*p* < 0.05).

The starch fractions of the LSCSs are shown in Figure 9. According to a component availability study of the *in vitro* digestion of starch, starch can be classified into RDS (< 20 min), SDS (20–120 min) and RS (> 120 min) in accordance with the time of starch digestion (33). The modulating effect of the cross-linking reaction on the digestive fraction of lotus seed starch may be clarified by measuring and calculating the variation in the difference between the contents of each fraction of the starch samples. As observed in the Figure 8, the RS content was significantly increased in the LSCSs compared with LS-0CS, and the RDS and SDS contents of the LSCSs decreased with the length of the cross-linking reaction, whereas the RS content increased accordingly. In the cross-linking reaction, the amorphous region was preferentially bound by the phosphorylating reagent, resulting in a change in the starch structure, decreases in the RDS and SDS contents and a corresponding increase in the RS content. Among the various cross-linking reaction products of starch, DSMP was reported to limit the enzymatic action of starch in the presence of a limited number of combined phosphorous and contributed to the formation of indigestible starch fractions (2). According to the phosphorous spectral analysis, the specific gravity of DMSP increased along with the degree of cross-linking. Consequently, the RS content of LS-12CS was the greatest among the samples. In summary, the enzymatic resistance of cross-linked RS of lotus seeds was influenced not only by solubility and swelling power, but also by structural properties, such as starch granule morphology and crystal structure.

## Conclusions

Different concentrations of cross-linking agents had significant effects on the structural properties and physicochemical properties of LSCSs, and the degree of cross-linking of LSCSs increased along with the addition of cross-linking agents. The cross-linked starches all exhibited appearances similar to the original starch, but the cross-linking agent played a “bridging” role, causing the granules to gradually aggregate, which resulted in more starch chains being “bound” together by the covalent bonds formed by the phosphate groups. The covalent bonds formed by the phosphate groups in the LSCSs were mainly composed of DMSP. The level of DMSP formed was correlated with the physicochemical properties of the LSCSs. Moreover, the cross-linking reaction mainly occurred in the amorphous regions. Compared with LS-0CS, the more complex and amorphous internal structures of LSCSs allowed the cross-linking reaction to raise the transition temperature, but lowered ΔH and the solubility, which was also related to the degree of LSCSs agglomeration. The RS increased along with the level of cross-linking, indicating a limitation for the digestive enzymes. The higher resistance of LS-12CS to digestion was not only related to its crystallinity, high DSMP specific gravity and other structural properties, but also to its low solubility. Based on its low solubility, heat resistance and high RS content, LS-12CS is a potential prebiotic for the food industry.

## Data availability statement

The original contributions presented in this study are included in the article/supplementary material, further inquiries can be directed to the corresponding authors.

## Author contributions

LL: writing the original draft, investigation, and methodology. SH and YL: conceptualization and software. BZ, YZ, and HZ: writing – review and editing and formal analysis. All authors contributed to the article and approved the submitted version.
